# Analysis of disulphide bond linkage between CoA and protein cysteine thiols during sporulation and in spores of *Bacillus* species

**DOI:** 10.1093/femsle/fnaa174

**Published:** 2020-11-18

**Authors:** Alexander Zhyvoloup, Bess Yi Kun Yu, Jovana Baković, Mathew Davis-Lunn, Maria-Armineh Tossounian, Naam Thomas, Yugo Tsuchiya, Sew Yeu Peak-Chew, Sivaramesh Wigneshweraraj, Valeriy Filonenko, Mark Skehel, Peter Setlow, Ivan Gout

**Affiliations:** Department of Structural and Molecular Biology, University College London, Gower St., London WC1E 6BT, UK; Department of Structural and Molecular Biology, University College London, Gower St., London WC1E 6BT, UK; Department of Structural and Molecular Biology, University College London, Gower St., London WC1E 6BT, UK; Department of Structural and Molecular Biology, University College London, Gower St., London WC1E 6BT, UK; Department of Structural and Molecular Biology, University College London, Gower St., London WC1E 6BT, UK; Department of Structural and Molecular Biology, University College London, Gower St., London WC1E 6BT, UK; Department of Structural and Molecular Biology, University College London, Gower St., London WC1E 6BT, UK; Biological Mass Spectrometry & Proteomics Cell Biology, MRC Laboratory of Molecular Biology, Francis Crick Avenue, Trumpington, Cambridge CB2 0QH, UK; Section of Microbiology, Faculty of Medicine and MRC Centre for Molecular Bacteriology and Infection, Imperial College London, Flowers Building, Imperial College Road, London SW7 2AZ, UK; Institute of Molecular Biology and Genetics, National Academy of Sciences of Ukraine, 150 Zabolotnogo St., Kyiv 03680, Ukraine; Biological Mass Spectrometry & Proteomics Cell Biology, MRC Laboratory of Molecular Biology, Francis Crick Avenue, Trumpington, Cambridge CB2 0QH, UK; Department of Molecular Biology and Biophysics, UConn Health, 263 Farmington Avenue, Farmington, CT 06030-3305, USA; Department of Structural and Molecular Biology, University College London, Gower St., London WC1E 6BT, UK; Institute of Molecular Biology and Genetics, National Academy of Sciences of Ukraine, 150 Zabolotnogo St., Kyiv 03680, Ukraine

**Keywords:** *Bacillus* species, oxidative stress, bacterial spores, sporulation, coenzyme A, protein CoAlation

## Abstract

Spores of *Bacillus* species have novel properties, which allow them to lie dormant for years and then germinate under favourable conditions. In the current work, the role of a key metabolic integrator, coenzyme A (CoA), in redox regulation of growing cells and during spore formation in *Bacillus megaterium* and *Bacillus subtilis* is studied. Exposing these growing cells to oxidising agents or carbon deprivation resulted in extensive covalent protein modification by CoA (termed protein CoAlation), through disulphide bond formation between the CoA thiol group and a protein cysteine. Significant protein CoAlation was observed during sporulation of *B. megaterium*, and increased largely in parallel with loss of metabolism in spores. Mass spectrometric analysis identified four CoAlated proteins in *B. subtilis* spores as well as one CoAlated protein in growing *B. megaterium* cells. All five of these proteins have been identified as moderately abundant in spores. Based on these findings and published studies, protein CoAlation might be involved in facilitating establishment of spores’ metabolic dormancy, and/or protecting sensitive sulfhydryl groups of spore enzymes.

## Teaser

Analysis of protein CoAlation during sporulation and in spores of *Bacillus* species.

## INTRODUCTION

Bacterial spores of Firmicute species are likely nature's most resilient life forms, and are extremely resistant to desiccation, pressure, chemicals, heat, cold, and radiation and can survive in unfavourable conditions for long periods (Setlow 2014; Paul *et al*. 2019). The molecular basis of sporulation in bacteria has been the subject of extensive studies, especially in *Bacillus* species. Once formed, spores are metabolically inert and can persist in the environment, immune to diverse biotic and abiotic stresses (Tan *et al*. 2015). A wealth of data is available on the genetics and biochemistry of sporulation, spore structure and how spores return to life in germination and outgrowth (Dworkin and Shah 2010; Setlow 2014; Setlow 2019).

Dormant bacterial spores are ‘preloaded’ with low energy molecules such as NAD^+^ and AMP, key compounds needed to generate ATP and NADH (Setlow and Setlow 1977a; Loshon and Setlow 1993). Another of these compounds is coenzyme A (CoA-SH, hereinafter referred to as CoA), a key metabolic integrator in all living organisms, not in an acylated form in spores but mostly disulphide bonded with protein cysteine residues (CoA-S-S-protein) (Setlow and Setlow 1977b). CoA's chemical structure allows this coenzyme to activate carbonyls to produce important thioester derivatives, such as acetyl-CoA (Theodoulou *et al*. 2014). Levels of CoA and the CoA/CoA thioester ratio in prokaryotic cells are tightly regulated by extracellular stimuli, nutrients, intracellular metabolites and stresses (Leonardi *et al*. 2005; Srinivasan and Sibon 2014), and CoA and its derivatives have well-established roles in cellular metabolism and gene regulation. CoA thioesters also are substrates for protein acylation, a modification important in regulating transcription, chromatin maintenance and metabolism in eukaryotes and prokaryotes (Chen *et al*. 2007; Lin, Su and He 2012; Choudhary *et al*. 2014).

One aspect of CoA's cellular functions that has not been well investigated is the role of this cofactor in thiol-disulphide exchange reactions and redox regulation. Recent studies have revealed a novel function of CoA in redox regulation, the covalent modification of cellular proteins by disulphide bond formation, termed CoAlation, in response to oxidative and metabolic stress (Tsuchiya *et al*. 2017, 2018). Protein CoAlation is a reversible and widespread protein modification, involving covalent attachment of CoA via its thiol group to reactive thiols of cysteine residues (CoA-S-S-protein) (Aloum *et al*. 2019; Tsuchiya *et al*. 2020). To study protein CoAlation, we have developed: (i) anti-CoA monoclonal antibodies; (ii) a mass spectrometry-based methodology for the identification of CoAlated proteins and (iii) *in vitro* CoAlation and deCoAlation assays (Malanchuk *et al*. 2015; Tsuchiya *et al*. 2017, 2018). These advances have identified over one thousand CoAlated proteins in prokaryotic and eukaryotic cells under various stress conditions (Gout 2018; Gout 2019).

In contrast to other low molecular weight (LMW) thiols, including glutathione, bacillithiol and mycothiol, CoA is produced in all living cells (Strauss 2010). In Gram-positive bacteria, which do not produce glutathione, CoA may function in antioxidant defence alongside bacillithiol and mycothiol (Imber, Pietrzyk-Brzezinska and Antelmann 2019). In this study, we demonstrate extensive protein CoAlation in *Bacillus megaterium* and *Bacillus subtilis* upon exposure of growing cells to oxidising agents and metabolic stress, and protein CoAlation increased significantly during *B. megaterium* sporulation, as seen previously (Setlow and Setlow 1977b). Protein CoAlation was also observed in spores of wild-type *B. subtilis* and a coat-defective mutant, and four abundant CoAlated proteins and sites of CoAlation were identified, as well as one highly CoAlated protein in growing *B. megaterium* cells. Possible roles of protein CoAlation in maintaining spores’ metabolic dormancy and resistance properties are discussed.

## RESULTS

### Protein CoAlation is induced in *B. megaterium* cells in response to oxidising agents or carbon starvation in a dose- and time-dependent manner

**Figure 1.**
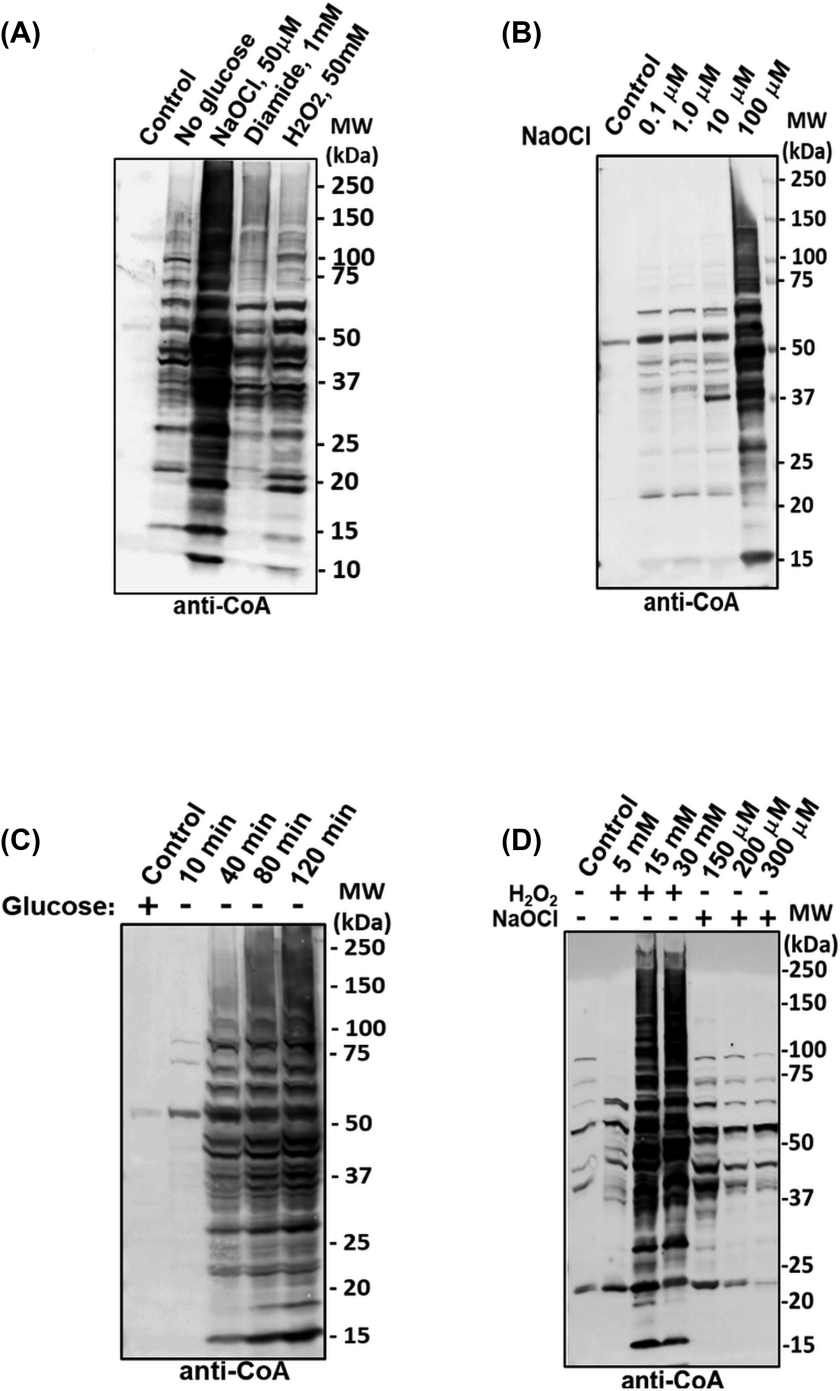
Protein CoAlation in *B. megaterium and B. subtilis cells* is induced by metabolic or oxidative stress. Exponentially growing *B. megaterium* cells were: **(A)**, treated with 50 μM NaOCl, 1 mM diamide or 50 mM H_2_O_2_ for 30 min or cultured in M9 medium without carbon source (glucose) for 40 min, **(B)** treated with increasing concentrations of NaOCl for 40 min or **(C)** harvested and suspended in M9 minimal medium without glucose. Harvested cells were lysed and the extracted proteins were separated by SDS-PAGE, followed by Western blotting with anti-CoA antibody. The blot shown is a representative from three independent repeats. **(D)**, Exponentially growing *B. subtilis* cells were treated with increasing concentrations of H_2_O_2_ or NaOCl for 30 min, proteins extracted, separated by SDS-PAGE and Western blotted with anti-CoA antibody.

To examine protein CoAlation in response to oxidising agents, growing *B. megaterium* cells were treated with hydrogen peroxide (H_2_O_2_), diamide or sodium hypochlorite (NaOCl). Western blotting with anti-CoA antibody revealed only several weak immunoreactive bands in the control sample, but extensive protein CoAlation in cells treated with oxidising agents (Fig. 1A). The strongest induction of protein CoAlation was with NaOCl and the pattern of immunoreactive bands differed somewhat in H_2_O_2_- or diamide-treated cells. Treatment of cells with increasing concentrations of NaOCl showed increased protein CoAlation in a dose-dependent manner (Fig. 1B). Importantly, incubation of extracts with DTT, a reducing agent, eliminated essentially all CoAlated proteins (data not shown), consistent with proteins being CoAlated through disulphide bond formation.

Transferring exponentially growing cells to organic carbon-free medium resulted in a time-dependent increase in protein CoAlation (Fig. 1A and C), compared to cells in rich medium, where only a single strongly CoAlated protein band of ∼55 kDa was observed (Fig. 1C; and see below). The pattern of CoAlated proteins in carbon-starved *B. megaterium* cells is different from that observed in response to oxidising agents (Fig. 1), indicating the involvement of different redox-sensing and response strategies under different cellular stress conditions. Overall, the results suggest that protein CoAlation in response to oxidative and metabolic stress is a dynamic and coordinated process.

### Oxidising agents induce protein CoAlation in *B. subtilis* cells

*Bacillus subtilis* is one of the best characterized Gram-positive bacteria, with a wealth of information on its response to chemical and physical stresses, including reactive oxygen species, oxidative damage and cellular defence mechanisms (Zuber 2009; Tran *et al*. 2019). However, the antioxidant function of CoA has not been investigated in *B. subtilis*. As shown in Fig. 1D, treatment of growing *B. subtilis* cells with H_2_O_2_ for 30 min resulted in a dose-dependent rise of protein CoAlation, with highest CoAlation with ∼15 mM H_2_O_2_. Increased protein CoAlation was also observed in *B. subtilis* cells treated with 150 μM NaOCl, while higher NaOCl concentrations showed a weaker effect, perhaps due to overoxidation of reactive cysteine residues to sulfinic or sulfonic states. Diamide also induced protein CoAlation in growing cells (data not shown).

### Protein CoAlation is greatly increased during *B. megaterium* sporulation

Under unfavourable growth conditions, many *Bacillus* species and their close relatives can form spores. Sporulation involves extensive alterations in the biochemistry, morphology and physiology of the sporulating cell and developing spore. Previous analysis of CoA and CoA thioesters in dormant and germinated spores of *B. megaterium* (Setlow and Setlow 1977b) found that while growing cells had high levels of acetyl-CoA, less than 1.5% of this CoA thioester was found in dormant spores which contained 32% of reduced CoA, 25% of oxidized CoA (CoA-S-S-CoA) and 43% of CoA in disulphide linkage to proteins (CoA-S-S-protein). High percentages of CoA were also in disulphide linkage to proteins in *Bacillus cereus* and *Clostridium bifermentans* spores (Setlow and Setlow 1977b). Most of these disulphides were cleaved during the first 15 min of spore germination, and <2% of CoA in stationary phase *B. megaterium* cells was disulphide linked to proteins.

**Figure 2.**
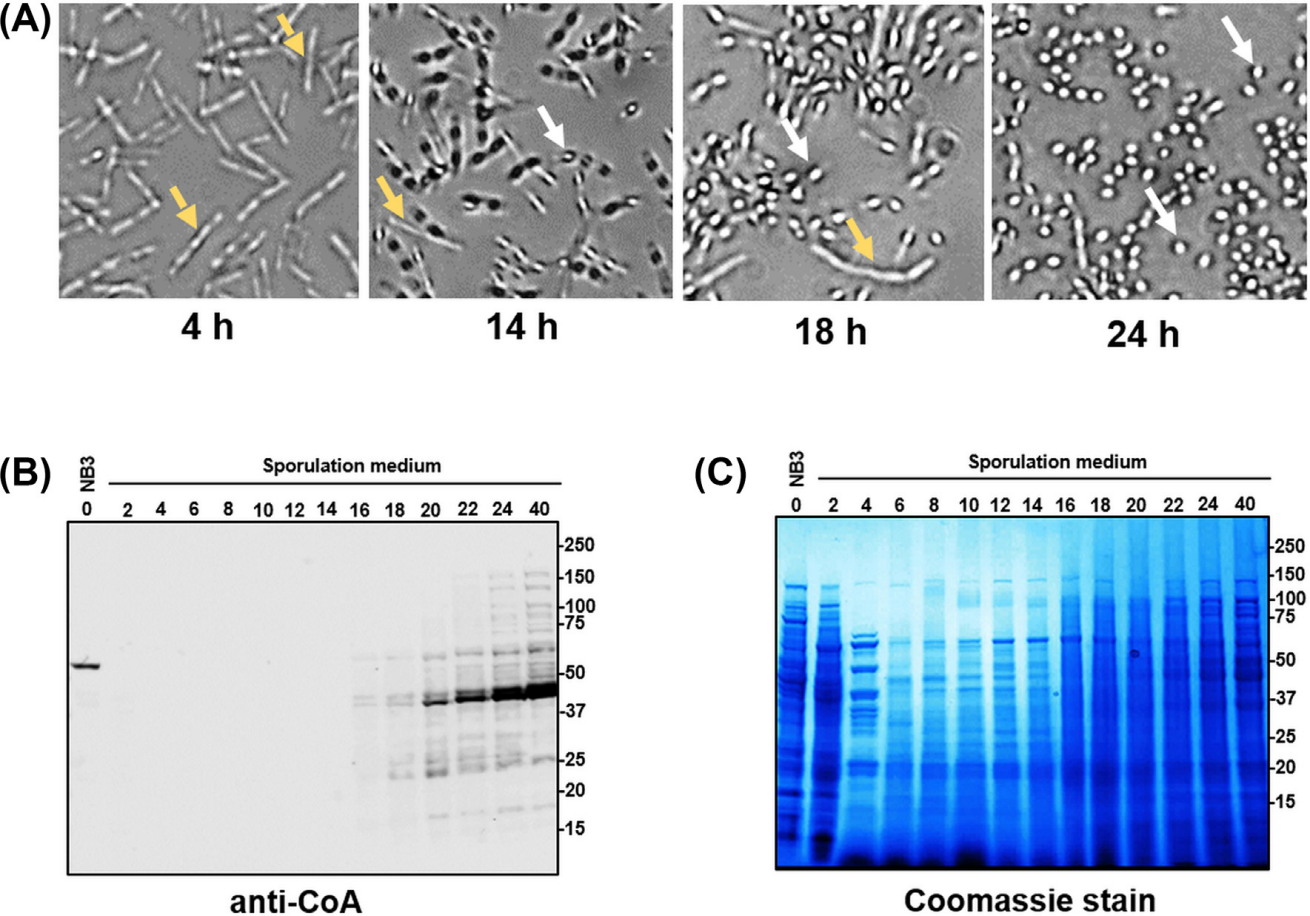
Analysis of protein CoAlation during *B. megaterium* sporulation. **(A)**, The progress of sporulation was assessed by phase-contrast microscopy at 40x magnification at various times after transfer of cells into a sporulation medium. The yellow and white arrows denote growing cells and phase-bright spores, respectively. **(B)**, Exponentially growing *B. megaterium* cells were transferred into sporulation medium and cultured for different time periods. Harvested cells were disrupted, extracted proteins separated by SDS-PAGE and Western blotted with anti-CoA antibody. The time 0 h sample is from cells cultured in NB3 medium prior to their transfer into sporulation medium. **(C)**, Coomassie staining of SDS-PAGE separated protein samples from (B). The Western blot analysis and sporulation time course are representative of three independent repeats of this experiment.

The work cited above also showed that high CoAlated protein levels appeared only late in *B. megaterium* sporulation and only in the developing spore, but technology available at that time was not able to readily identify specific CoAlated proteins. However, recent development of anti-CoA antibodies (Malanchuk *et al*. 2015) allowed examination of individual CoAlated proteins during *B. megaterium* sporulation (Fig. 2). Analysis of the sporulation efficiency by phase-contrast microscopy revealed that levels of phase-bright spores in cells were: <3% at 14 h, 12% at 18 h and 85% at 24 h (Fig. 2A). These findings show that: (i) the majority of *B. megaterium* cells sporulated by 24 h and (ii) the appearance of phase-bright spores corresponded with the increased CoAlation of spore proteins. The latter is important, since shutdown of metabolism in developing spores parallels the appearance of phase-bright spores, including the disappearance of forespore NADH and NADPH (Setlow and Setlow 1977a,b).

Analysis of levels of CoAlated proteins in growing and sporulating *B. megaterium* cells (Fig. 2B) found only one ∼55 kDa protein, which was significantly CoAlated in exponentially growing cells (0 h), but this CoAlated protein disappeared 2 h later. Mass spectrometric analysis (Fig. 4A) identified the CoAlated peptide in the protein 3-bisphoshoglycerate-independent phosphoglycerate mutase (iPGM–56 kDa), which is a cofactor-independent and Mn^2+^-dependent enzyme that catalyses the isomerisation of 2- and 3-phosphoglycerates (2PGA and 3PGA). The activity of iPGM is essential in *Bacillus* cells growing on glucose and the regulation of this enzyme's activity is particularly important during sporulation and spore germination of *Bacillus* species (Singh and Setlow 1979a).

Several immunoreactive bands appeared 16 h after transfer to the sporulation medium, and immunoreactive bands increased gradually, reaching almost peak levels at 24 h (Fig. 2B). The gradual increase in protein CoAlation correlated well with the gradual increase of spore formation observed by phase-contrast microscopy, and analysis of the same samples by Coomassie blue staining revealed significant changes in the protein pattern, especially soon after transfer of cells to sporulation medium (Fig. 2C).

### Analysis of protein CoAlation in *B. subtilis* spores

**Figure 3.**
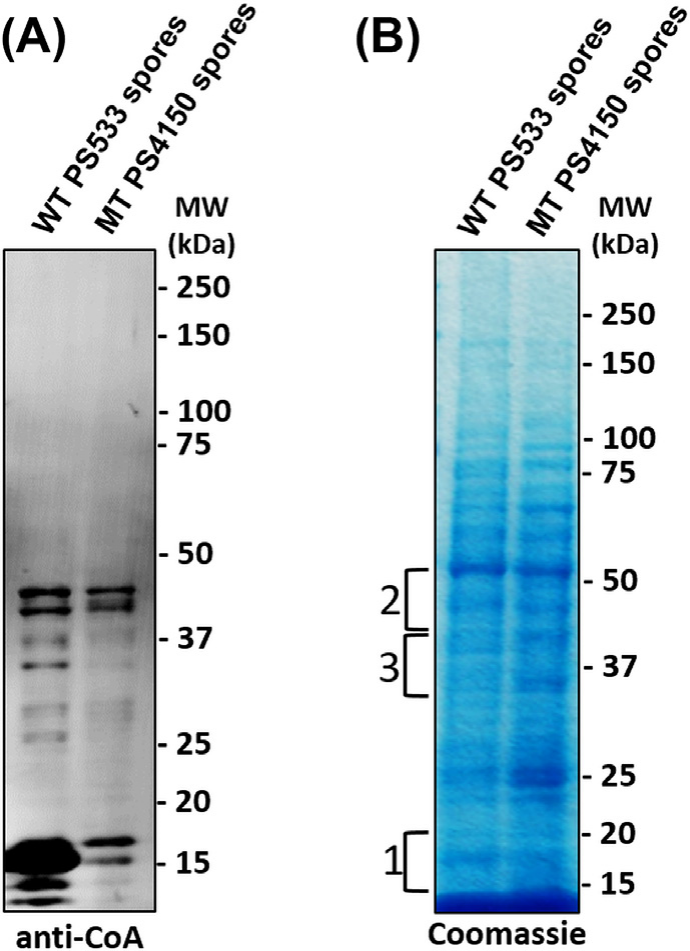
Analysis of protein CoAlation in *B. subtilis* spores. Proteins were extracted from spores of *B. subtilis* wild-type (WT PS533) and its isogenic mutant (MT PS4150), which lacks the *cotE* and *gerE* genes essential for spore coat assembly. The samples were separated by SDS-PAGE under non-reducing conditions and Western blotted with anti-CoA mAb **(A)** or Coomassie Blue stained **(B)**. The numbered brackets on the left indicate the regions from which gel bands were excised and analysed via mass spectrometry.

The increased protein CoAlation in developing spores during *B. megaterium* sporulation made it of obvious interest to examine CoAlated proteins in spores, with the goal of ultimately identifying these proteins. However, the *B. megaterium* spore proteome has not been determined. This prompted us to examine CoAlated proteins in *B. subtilis* spores, as the *B. subtilis* genome is well-annotated as is its spore proteome, and mutations in almost all *B. subtills* genes are available (Kunst *et al*. 1997; McKenney, Driks and Eichenberger 2013; Swarge *et al*. 2018; Zhu and Stülke 2018). Consequently, we used purified spores of *B. subtilis* strains: (i) PS533, the wild-type strain and (ii) PS4150, an isogenic mutant lacking the *cotE*and *gerE* genes essential for spore coat assembly (Ghosh *et al*. 2008). PS4150 spores lack almost all coat proteins, and since these comprise a large amount of total spore protein, with >80 different coat proteins (Driks and Eichenberger 2016), these spores’ use might simplify analysis of CoAlated proteins in the spores’ core. Spores were ruptured by mechanical breakage in the presence of N-ethylmaleimide (NEM), and the breakage as assessed by phase-contrast microscopy was >95% (data not shown). Analysis by Western blotting of CoAlated proteins in these extracts revealed a number of immunoreactive bands from spores of both strains (Fig. 3A). However, the pattern of protein CoAlation was different in wild-type and mutant spores. The most striking difference was the several highly CoAlated bands between 10 kDa and 17 kDa in wild-type spores, which were not at as high levels in mutant spores; perhaps these proteins were coat or coat-associated proteins (see Discussion). There were also several higher molecular weight immunoreactive bands, some of which were at similar levels in both coat-defective and wild-type spores (Fig. 3A).

**Figure 4.**
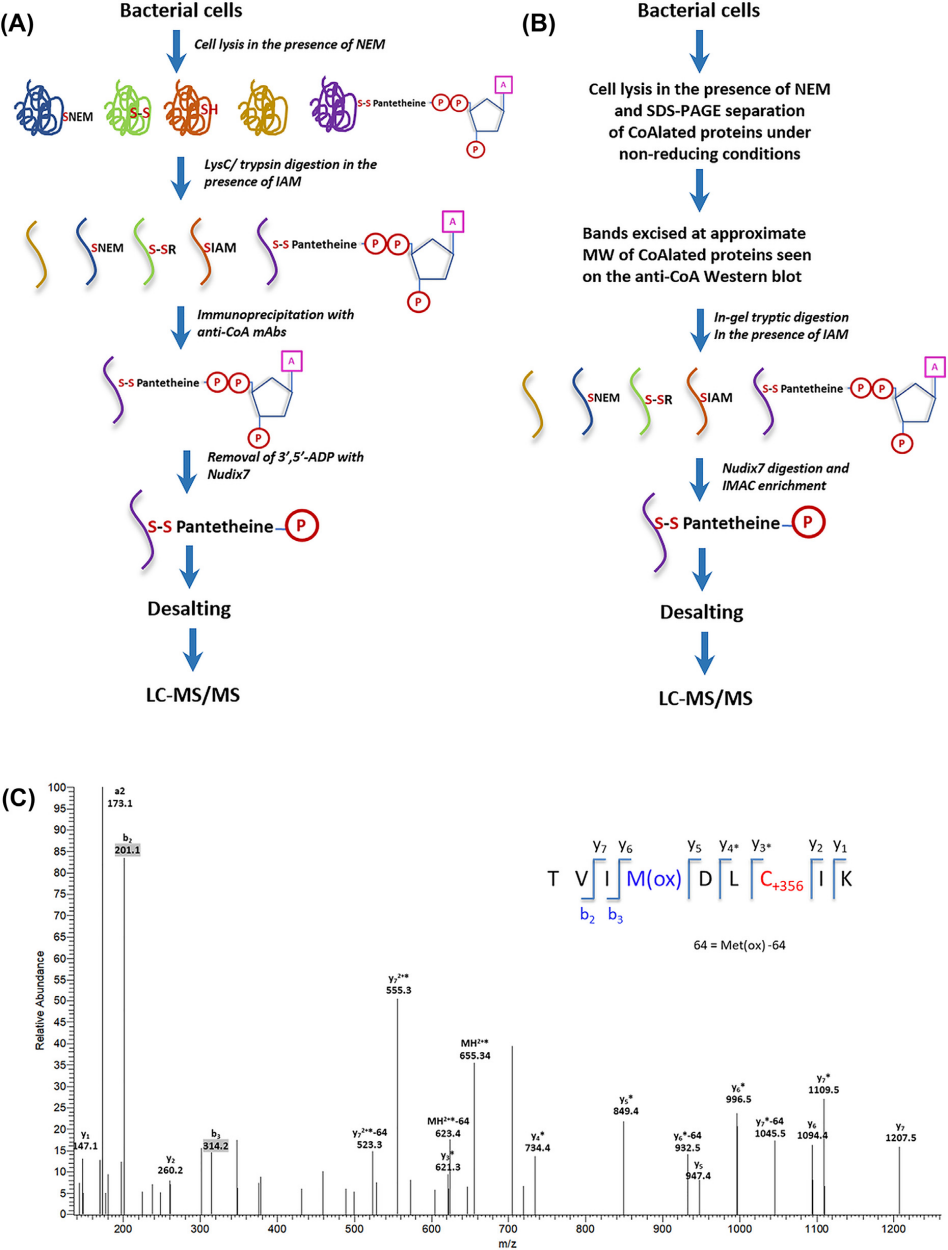
Identification of CoAlated peptides using LC-MS/MS. Schematic diagrams showing the steps involved in sample preparation and the identification of CoAlated peptides by LC-MS/MS from bacterial cell lysates **(A)** and excised gel slices **(B)**. **(C)** Annotated LC-MS/MS spectrum of a CoAlated peptide, YneT from *B. subtilis* spores. The MS/MS spectrum was obtained as described in Methods and shows the peptide (TVIMDL**C^126^**IK) corresponding to YneT, containing CoA-modified cysteine residues (C+356 corresponds to the 4-phosphopantetheine (4-PP)). Fragment ions are coloured blue and red for b- and y-ions, respectively. The asterisk (*) denotes the loss of phosphoric acid (−98 Da) from the precursor and/or product ions that contained the CoA-modified cysteine residue, while the loss of 64 Da corresponds to the loss of methanesulfonic acid from the oxidised methionine side chain (Met(ox)-64).

To identify the heavily CoAlated proteins in wild-type spores, and to locate the sites of CoAlation, three slices corresponding to the location of anti-CoA immunoreactive bands on the Western blot (slice 1 (∼12–22 kDa); slice 2 (∼44–55 kDa); and slice 3 (∼30–44 kDa)) were excised from the stained gel (Fig. 3B) and subjected to digestion, treatment with Nudix7 to remove the 3-phosphoAMP and enrichment with an IMAC column before LC-MS/MS analysis (Fig. 4B). This analysis identified CoAlated peptides from 4 proteins—YneT, the H1 homolog of the thiol-specific peroxidase AhpC-H1, alcohol dehydrogenase AdhB and phosphopentomutase Drm (Fig. 4C and Table 1). Notably, all of these CoAlated proteins have been identified in the *B. subtilis* spore proteome (Swarge *et al*. 2018). YneT is of particular interest as its molecular weight is in the range of the multiple CoAlated spore proteins at much lower levels in spores that lack most coat proteins.

**Table 1.** Identification and properties of major CoAlated proteins in *B. subtilis* spores and sites of CoAlation. Four CoAlated proteins (YneT (Q45065), AhpC (P80239), Drm (P46353) and AdhB (O06012)) were identified by mass spectrometry. Their molecular weight (MW), activity and function are shown within this table. The position of CoA-modified cysteine residues in each protein sequence is shown and numbered.

Protein	CoAlated peptides	Predicted MW	Activity	Function	Reference
YneT (Q45065)	TVIMDLC^126^IK	135aa 15 kDa	Unknown	Putative CoA-binding protein	Rose & Entian (1996)
AhpC (P80239)	QNPGEVC^166^PAK	187aa 21 kDa	Thiol-specific peroxidase	Reduction of lipid hydroperoxides	Cha *et al*. (2015)
Drm (P46353)	DC^233^GLDVISIGK	394aa 44 kDa	Phosphopento-mutase	Nucleotide metabolism	Schuch *et al*. (1999)
AdhB (O06012)	IPESC^150^EEPDEK	378aa 41 kDa	Zinc-type alcohol dehydrogenase	Reversible oxidation of alcohols to acetaldehydes	Nguyen *et al*. (2009)

## DISCUSSION

A diverse range of mechanisms are used by bacteria to sense and combat oxidative stress, and to repair the resulting damage. The most effective defence mechanisms include antioxidant enzymes and LMW thiols including glutathione, bacillithiol and mycothiol, and now CoA. Analysis of CoAlated proteins in mammalian cells and bacteria exposed to oxidative or metabolic stress showed that over 65% of CoAlated proteins are involved in metabolism (Tsuchiya *et al*. 2017, 2018). Many metabolic enzymes and their regulators use cysteine residues for catalysis and redox regulation, and previous studies have shown that *in vitro* CoAlation of key metabolic enzymes inhibits their catalytic activity in a reversible DTT-dependent manner (Tsuchiya *et al*. 2017, 2018; Tsuji, Yoon and Ogo 2019).

In this study, Western blotting demonstrated extensive CoAlation of many proteins in growing *B. megaterium* and *B. subtilis* cells exposed to oxidising agents or nutrient deprivation, as well as during sporulation and in pure spores. A single CoAlated protein of ∼55 kDa was also observed in *B. megaterium* growing cells prior to induction of sporulation. We identified this protein as iPGM, a Mn^2+^-dependent phosphoglycerate mutase, the activity of which is regulated by levels of Mn^2+^ and pH changes (Singh and Setlow 1979a; Kuhn *et al*. 1995). In the developing forespore of *Bacillus* species, iPGM is inactive in developing spores’ core due to its low pH of ∼6.5 and this leads to accumulation of a large depot of 3PGA in the spore. However, soon after spore germination is triggered, spore core pH rises to ∼7.8, iPGM becomes active and the resultant conversion of 3PGA to 2PGA leads to generation of ATP which is important in the early minutes of spore outgrowth (Setlow and Kornberg 1970). Notably, this iPGM's activity is also abolished by sulfhydryl agents (Singh and Setlow 1979b). However, further studies are needed to understand the functional relevance of iPGM's modification by CoA in *Bacillus* cell's growth, sporulation and spore germination.

We identified four CoAlated proteins in wild-type *B. subtilis* spores. AdhB, an alcohol dehydrogenase (Nguyen *et al*. 2009) and AhpC, an alkyl hydroperoxide reductase, have been identified in the dormant spore core, and *adhB* is expressed in the developing spore (Casillas-Martinez and Setlow 1997; Zhu and Stülke 2018). Drm is a phosphopentosemutase (Schuch *et al*. 1999) involved in nucleotide metabolism, and as a metabolic enzyme, it is likely also located in the spore core, but this has not been shown directly. Of particular interest is YneT, as: (i) CoAlation of this protein appeared reduced in coat-defective spores and (ii) the structure of this protein appears to contain a CoA binding pocket, although no function for this protein has been ascribed (Rose and Entian 1996; Zhu and Stülke 2018). The specific function of these four proteins in spores is not clear, as only the effects of *ahpC* deletion on spore properties have been studied (Casillas-Martinez and Setlow 1997), and loss of AhpC had no effects on spores’ high resistance to alkyl hydroperoxides, consistent with this enzyme's inactivity in the dormant spore. Whether this inactivity is due to CoAlation of the enzyme's reactive thiol group or spores’ low water content (Cowan *et al*. 2003), or both, remains to be studied. Expression of AhpC in *B. subtilis* cells is induced by organic peroxides, suggesting the role of this peroxidase as an antioxidant scavenger of lipid hydroperoxides (Cha *et al*. 2015). In *S. aureus* (Sa), CoAlation of AhpC at Cys168 (equivalent to Cys166 in *B. subtilis*) was detected in diamide-treated growing cells (Tsuchiya *et al*. 2018). The effect of Cys168 CoAlation on Sa-AhpC activity has not been examined, but inhibition of its peroxidase activity seems likely. Perhaps AhpC CoAlation at Cys166 protects this crucial thiol group, so that enzyme activity can be restored by deCoAlation during germination.

This study raises important questions regarding the functional consequences of protein CoAlation and the regulation of CoAlation/deCoAlation in sporulation, dormant spores and germination. Protein CoAlation may occur via non-enzymatic and enzymatic reactions. The CoA pKa value is relatively high (9.83), which protects its thiol group from conversion to the sulfenic acid state under oxidative stress (Keire, Robert and Rabenstein 1992). Thus, for nucleophilic attack, the pKa of the CoA thiol has to be decreased. By analogy to protein glutathionylation and the role of glutathione transferase in this modification (Townsend *et al*. 2009), we propose that the enzymatic mechanism of protein CoAlation may involve a CoA transferase whose identification in prokaryotic and eukaryotic cells remains to be achieved.

Non-enzymatic protein CoAlation occurs via a disulphide exchange reaction (Tsuchiya *et al*. 2017, 2018). The majority of protein cysteinyl thiols have relatively high pKa values, and therefore, possess low deprotonation ability (Jensen *et al*. 2014). However, cysteinyl residues within a basic environment exhibit a lower pKa, and therefore, are more susceptible to deprotonation and thus oxidation to sulfenic acid. Since sulfenic acid is highly reactive, it has to be protected from overoxidation to sulfinic or sulfonic states, in particular by intermolecular disulphide bond formation with LMW thiols, including CoA.

Protein CoAlation is a reversible modification and the activity of two antioxidant enzymes is required to mediate efficient protein deCoAlation: (i) CoAredoxins or deCoAlases and (ii) CoA disulphide reductase (CoADR). CoAredoxin activity has been detected in bacteria and mammalian cells (Tsuchiya *et al*. 2017, 2018), but their identification is yet to be reported (Tossounian *et al*., unpublished data). CoADR activity was identified in *B. megaterium* spores (Swerdlow and Setlow 1983), and the gene for this enzyme was cloned from a variety of bacteria, including *Bacillus* species (delCardayre and Davies 1998; Wallen *et al*. 2008; Lencina *et al*. 2019). As CoA-modified proteins were shown to be very rapidly deCoAlated soon after initiation of spore germination when NADH and NADPH are generated (Setlow and Setlow 1977a, 1977b), CoAredoxin and CoADR are presumably present in spores and maintained in inactive states.

A second major question is what is the relevance of protein CoAlation in dormant bacterial spores? It has been shown that protein CoAlation protects them from irreversible sulfhydryl overoxidation (Tsuchiya *et al*. 2017, 2018, 2020). We speculate that reversible covalent modification of catalytic/regulatory cysteine residues of key players in metabolic pathways may contribute to the onset and maintenance of the metabolic dormancy and spore resistance.

It is also important to note that protein CoAlation is not confined to sporulation in bacteria, as levels of CoAlated proteins also increase markedly in the lower eukaryote, *Dictyostelium discoideum* upon fruiting body and spore formation (Aloum *et al*. 2019). This suggests that protein CoAlation might be a widespread phenomenon associated with metabolic dormancy, perhaps occurring in spores of other organisms, including fungi, algae, plants and bacterial spores of non-Firmicutes.

## MATERIALS AND METHODS

### Reagents and chemicals

Common chemicals were obtained from Sigma-Aldrich and Thermo Fisher Scientific unless otherwise stated. Production and characterization of the anti-CoA monoclonal antibody was as described (Malanchuk *et al*. 2015).

### Bacterial growth conditions and treatments

Cells of *B. megaterium* NCTC10342 and *B. subtilis* PS533 (Ghosh *et al*. 2008), a 168 strain (PS832), carrying plasmid pUB110 providing kanamycin resistance (10 µg mL^−1^), were grown at 37°C in Nutrient Broth 3 (NB3) medium (Sigma-Aldrich). To induce oxidative or metabolic stress, overnight cultures were diluted 1:100 in the same medium and grown to an optical density at 600 nm (OD_600_) of 0.7. *Bacillus megaterium* cells were then treated with or without oxidising agents for 30 min at 37°C. Growing cells were also treated with increasing concentrations of NaOCl for 40 min. Metabolic stress was induced by suspending growing *B. megaterium* cells at an OD_600_ of 0.7 in M9 medium (Sigma-Aldrich) lacking organic carbon, incubation at 37°C and sampling at various times. Oxidative stress was induced in *B. subtilis* cells by exposing growing cells at an OD_600_ of 0.7 prepared as described above to H_2_O_2_ or NaOCl.

### Induction of *B. megaterium* sporulation

An overnight *B. megaterium* culture was diluted 1:100 and grown at 37°C in NB3 medium to an OD_600_ of ∼0.7. Cells were collected by centrifugation (20 min, 3000 × *g*), suspended at an OD_600_ of ∼0.4 in Schaeffer's Sporulation Medium (SSM) (Schaeffer, Millet and Aubert 1965) at 37°C, and cultures were then incubated at 37°C with intensive shaking. At various times 2 mL aliquots were removed, collected cells rapidly chilled on ice with 25 mM NEM, harvested by centrifugation as described above, and pellets were frozen on dry ice and stored at −80°C. Sporulation efficiency was assessed using phase-contrast microscopy.

### Preparation of spores from *B. subtilis* PS533 and PS4150 strains

Spores of *B. subtilis* strains were prepared on 2 × Schaeffer's glucose medium agar plates (Nicholson and Setlow 1990), and harvested and purified, all as described previously (Setlow 2019). Phase-contrast microscopy was used to demonstrate that purified spores were >98% free of sporulating cells, germinated spores, and debris. Purified spores were lyophilised and stored at 4°C.

### Extraction of bacterial proteins

For the analysis of protein CoAlation in response to oxidising agents or carbon starvation, 3 mL samples of *B. megaterium* and *B. subtilis* cells were collected, chilled on ice with 25 mM NEM and centrifuged (5 min, 20 000 × *g*) at 4°C. Cells were ruptured in 100 µL Lysis buffer (50 mM Tris-HCl pH 7.5, 50 mM NaCl, 5 mM EDTA, 25 mM NEM, 0.1 mg mL^−1^ lysozyme and a protease inhibitor cocktail (Roche)). The lysate was incubated on ice for 20 min, mixed with an equal volume of 2% SDS, sonicated on ice to reduce viscosity and centrifuged (20 min, 20 000 × *g*) at 4°C. The supernatant was mixed with 1x non-reducing sample buffer and heated at 95°C for 5 min.

For analysis of protein CoAlation during sporulation, samples collected at various times were harvested and mixed with 10 μL of Lysis Buffer per OD_600_ of harvested cells, and incubated on ice for 20 min. The suspension was mixed with an equal volume of 2% SDS and two volumes of zirconia/silica beads (0.1 mm, BioSpec Products, Cat# 11079101z) and processed on a TissueLyser II for mechanical rupture at 30 Hz for 10 min. Samples were mixed with 1x non-reducing sample buffer and incubated at 95°C for 10 min, heat-treated samples centrifuged at 20 000 ×   *g* for 20 min and the supernatant used for bacterial protein separation by SDS-PAGE and Western blotting with anti-CoA antibodies (Tsuchiya *et al*. 2017).

### Mass spectrometry of CoAlated proteins

Lyophilised *B. subtilis* spores were harvested and disrupted to extract proteins as described above for *B. megaterium* sporulating cells. Separation of protein lysates by SDS-PAGE was carried out in duplicate, one for Western blot analyses and one for Coomassie staining. Gel slices corresponding to the locations of anti-CoA immunoreactive bands (Fig. 3) were excised from the stained gel. In-gel digestion with sequencing grade trypsin (Promega) was carried out at 37°C overnight, and peptides were treated with Nudix7 and enriched by an IMAC column before LC-MS/MS analysis (Fig. 4B) as previously described (Tsuchiya *et al*. 2017). LC-MS/MS raw data files were processed as standard samples using MaxQuant version 1.5.2.8 (Cox and Mann 2008) and searched against the *B. subtilis* (strain 168) UniProt protein database.

To identify the ∼55 kDa CoAlated protein in exponentially growing *B. megaterium* cells, cells were harvested at OD_600_ of 0.7, lysed in the presence of NEM, and CoAlated proteins were identified by mass spectrometry as previously described by (Tsuchiya *et al*. 2017). Schematic diagrams of the LC-MS/MS-based methodology for the identification of CoAlated proteins from cell lysates and gel slices are shown in Figs 4A and B, respectively.
